# Non-Invasive Urine Test Predicts Grade Group Upgrading in Patients on Active Surveillance for Prostate Cancer: Multisite Validation and Comparison with MRI

**DOI:** 10.1097/JU.0000000000005095

**Published:** 2026-04-28

**Authors:** Jeffrey J. Tosoian, Jacob I. Meyers, Bradley Moore, Cameron J. Britton, Kent Chevli, Walter Rayford, Adam J. Gadzinski, Jean V. Joseph, Pratik Patel, Ali Kasraeian, Prithipal S. Sethi, Hunter S. Robinson, Keavash D. Assani, Vijay Rings, Janene Pierce, Kristen R. Scarpato, Kelvin A. Moses, Daniel A. Barocas, Sam S. Chang, David F. Penson, Martin G. Sanda, Ashley E. Ross, Matthew R. Cooperberg, Keyan Salari, Michael Liss, Yuping Zhang, Javed Siddiqui, Udit Singhal, Simpa S. Salami, Todd M. Morgan, Ganesh S. Palapattu, John T. Wei, Jingyi Cao, Vikram Bala, Younggook Oh, Spencer Heaton, Yingye Zheng, Arul M. Chinnaiyan

**Affiliations:** Department of Urology, Vanderbilt University Medical Center; Vanderbilt-Ingram Cancer Center, Nashville, TN; Lynx Dx, Ann Arbor, MI; Lynx Dx, Ann Arbor, MI; Department of Urology, Vanderbilt University Medical Center, Nashville TN; Western New York Urology Associates, Cheektowaga, NY; The Urology Group, Memphis, TN; Comprehensive Urology, Royal Oak, MI; Department of Urology, University of Rochester Medical Center, Rochester, NY; Arizona State Urological Institute (ASUI) and Arizona State Urological Research Institute (ASURI), Gilbert, AZ; Kasraeian Urology, Jacksonville, FL; Golden State Urology, Sacramento, CA; Geisinger Medical Center, Department of Urology, Danville, PA; Department of Urology, Vanderbilt University Medical Center; Nashville, TN; Department of Urology, Vanderbilt University Medical Center; Nashville, TN; Department of Urology, Vanderbilt University Medical Center; Nashville, TN; Department of Urology, Vanderbilt University Medical Center; Nashville, TN; Department of Urology, Vanderbilt University Medical Center; Nashville, TN; Department of Urology, Vanderbilt University Medical Center; Vanderbilt-Ingram Cancer Center, Nashville, TN; Department of Urology, Vanderbilt University Medical Center; Vanderbilt-Ingram Cancer Center, Nashville, TN; Department of Urology, Vanderbilt University Medical Center; Vanderbilt-Ingram Cancer Center, Nashville, TN; Department of Urology, Emory University School of Medicine, Atlanta, GA; Northwestern University Department of Urology, Chicago, IL; University of California San Francisco Departments of Urology and Epidemiology & Biostatistics, San Francisco, CA; Department of Urology, Massachusetts General Hospital, Harvard Medical School, Boston, MA; Department of Urology, UC San Diego Health, San Diego, CA; Department of Pathology, University of Michigan, Ann Arbor, MI; Department of Pathology, University of Michigan, Ann Arbor, MI; Department of Urology, University of Michigan, Ann Arbor, MI Rogel Cancer Center, Ann Arbor, MI; Department of Urology, University of Michigan, Ann Arbor, MI Rogel Cancer Center, Ann Arbor, MI; Department of Urology, University of Michigan, Ann Arbor, MI Rogel Cancer Center, Ann Arbor, MI; Department of Urology, University of Michigan, Ann Arbor, MI Rogel Cancer Center, Ann Arbor, MI; Department of Urology, University of Michigan, Ann Arbor, MI Rogel Cancer Center, Ann Arbor, MI; Lynx Dx, Ann Arbor, MI; Lynx Dx, Ann Arbor, MI; Lynx Dx, Ann Arbor, MI; Lynx Dx, Ann Arbor, MI; Fred Hutchinson Cancer Research Center; Departments of Pathology and Urology, University of Michigan, Ann Arbor, MI; Rogel Cancer Center, Ann Arbor, MI; Howard Hughes Medical Institute, Chevy Chase, MD

**Keywords:** Prostate cancer, biomarkers, biopsy, magnetic resonance imaging, liquid biopsy

## Abstract

**Purpose:**

We developed and externally-validated a non-DRE urine test to inform whether biopsy is necessary in patients undergoing active surveillance (AS). Performance and clinical consequences of testing were directly compared to multiparametric magnetic resonance imaging (mpMRI).

**Materials and Methods:**

Biomarker models (MyProstateScore 2.0 - Active Surveillance [MPS2-AS]) were derived to predict upgrading to Grade Group (GG)≥3 and GG≥2 and externally validated across 11 practices. Urine was prospectively collected, and patients underwent ≥12-core systematic biopsy (SBx) plus targeted biopsy (TBx) of PI-RADS≥3 lesions. Diagnostic performance and clinical consequences of urinary testing to determine the need for biopsy were compared to mpMRI.

**Results:**

The validation cohort included 330 patients with GG1 cancer scheduled for AS biopsy. Overall, 280 (85%) patients had pre-biopsy mpMRI, of which 130 (46%) were PI-RADS 1-2, 42 (15%) were PI-RADS 3, and 108 (39%) were PI-RADS 4-5. On biopsy, 31 (9.4%) patients upgraded to GG≥3 and 123 (37%) to GG≥2. MPS2-AS provided higher AUC than mpMRI for upgrading to both GG≥3 (0.82 vs. 0.73) and GG≥2 (0.74 vs. 0.64). Clinically, pre-biopsy MPS2-AS would have avoided 64% of unnecessary biopsies while failing to detect only 3.2% of GG≥3 upgrades. By contrast, use of PI-RADS≥3 would have failed to detect 18% of GG≥3 upgrades and avoided fewer unnecessary biopsies (50%). Performance of MPS2-AS was consistent across clinically-pertinent subgroups (confirmatory and surveillance biopsy, Black and non-Black patients).

**Conclusions:**

In a multisite AS population, urinary MPS2-AS provided highly accurate and actionable testing for GG≥3 and GG≥2 upgrading, meaningfully outperforming mpMRI on direct comparison. These findings suggest that non-invasive monitoring with MPS2-AS could reduce the need for scheduled biopsies and serial mpMRI.

## INTRODUCTION

Active surveillance (AS) is the preferred management option for low-risk prostate cancer (PCa).^1–3^ AS aims to reduce overtreatment of cancers that pose minimal risk of harm while monitoring for higher-grade tumors that necessitate treatment. Supported by data reflecting excellent long-term oncologic outcomes,^4^ acceptance of AS has increased substantially, with nearly 60% of patients with Grade Group 1 (GG1) cancers now managed on AS.^5, 6^

AS monitoring protocols have traditionally included serial prostate-specific antigen (PSA) testing, digital rectal examination (DRE), and, more recently, multiparametric magnetic resonance imaging (mpMRI) to monitor for evidence of cancer upgrading.^1–3^ Yet each of these modalities comes with notable limitations. PSA kinetics do not reliably predict grade reclassification on AS,^7^ and the limited accuracy of DRE is well-described.^8^ While informative in the diagnostic setting, several expert centers have shown that mpMRI lacks accuracy to reliably rule-out or rule-in upgrading in AS patients.^9–11^ A recent meta-analysis revealed that mpMRI missed 41% of higher-grade cancers during surveillance.^12^ Based on the limitations of these tools, repeat biopsies remain a required component of AS, despite associated discomfort and increasing risk of complications over time.^13^

A major priority in the field is to reduce the need for invasive testing during AS while maintaining excellent oncologic outcomes.^14^ While existing biomarker tests have shown an association with cancer upgrading,^15, 16^ none have provided a clear clinical approach to reliably inform the need for forthcoming surveillance biopsies. MyProstateScore 2.0 (MPS2) is an 18-gene urinary test validated to inform biopsy decision-making in patients with elevated PSA.^17^ Because MPS2 captures genes uniquely overexpressed in high-grade versus low-grade cancers, there is a strong biologic foundation supporting its ability to differentiate between AS patients truly harboring only low-grade disease and those most likely to harbor occult high-grade cancers.

In the current study, we developed an MPS2-based non-DRE testing approach (MyProstateScore 2.0 - Active Surveillance [MPS2-AS]) to inform the need for repeat biopsy during AS, externally validated this approach in a prospectively-enrolled multisite cohort, and directly compared the accuracy of MPS2-AS testing with mpMRI in the AS population.

## MATERIALS AND METHODS

Institutional review board approval was obtained from the Western Institutional Review Board-Copernicus Group (20241020) and site IRBs (UM-HUM00037879, VUMC220357), and all participants provided written informed consent. This study followed the Transparent Reporting of a multivariable prediction model for Individual Prognosis Or Diagnosis (TRIPOD) Statement and Standards for Reporting of Diagnostic Accuracy (STARD) guideline.^18, 19^

### Model Development

The development cohort included 761 patients presenting for diagnostic biopsy (Supplementary Table 1) at the University of Michigan as previously described^17^ and detailed in the Supplement. Prebiopsy urine was prospectively-collected under an IRB-approved National Cancer Institute protocol. RNA isolation, extraction, and complementary DNA synthesis were performed as previously described.^17^ Biomarker amplification and measurement were performed using the QuantStudio 12K Flex Real-Time PCR System (Thermo Fisher Scientific).^17^ Biomarker expression was quantified by the relative cycle threshold (Ct), defined as the number of amplification cycles required for fluorescence to exceed background. All samples were run in triplicate, and mean Ct values were normalized to the prostate-specific reference gene *KLK3*. Normalized mean Ct values were used for analyses.

We developed distinct models to predict cancer upgrading from GG1 to GG≥3 (MPS2-AS GG1-3 model) and GG1 to GG≥2 (MPS2-AS GG1-2 model) as detailed in the Supplement. In brief, optimal combinations of predictive genes were identified from the MPS2 18-gene panel using recursive feature elimination and coefficient-based filtering. PSA density (PSAD) was locked into models *a priori* based on predictive value for high-grade cancer.^20^ Model outputs were calibrated to provide the percentage risk (0-100%) of upgrading to GG≥3 and GG≥2. In accordance with principles of diagnostic biomarker testing from the National Comprehensive Cancer Network,^21^ MPS2-AS thresholds were set to achieve a sensitivity ≥90% and specificity ≥30% for higher-grade cancer. Thresholds were calibrated to equal 11.5%, consistent with MPS2 non-DRE diagnostic testing.^22^

The locked MPS2-AS models for upgrading to GG≥3 and GG≥2 incorporated 5 genes and 9 genes from the MPS2 testing panel, respectively (Supplementary Table 2). The MPS2-AS-based predicted probabilities of upgrading aligned closely with the observed prevalence of upgrading, indicating good calibration (Supplementary Figure 1).

### Power Calculation

We performed a formal power analysis using prospective-specimen-collection, retrospective-blinded-evaluation (PRoBE) principles for a rule-out test using a continuous biomarker.^23^ Validation cohort sizes exceeded the calculated sample sizes required (Supplementary Table 3), confirming the current analysis was sufficiently powered.

### Prospective Multisite Validation

A total of 1,381 patients enrolled in the prospective biospecimen protocol at 11 urology practices (Supplementary Figure 2). The current analysis included 330 patients with GG1 PCa that were undergoing confirmatory or surveillance biopsy as part of their planned clinical management (i.e. the decision to pursue biopsy was made prior to and independent of enrollment). Patients taking 5-alpha reductase inhibitors were excluded. Most patients underwent pre-biopsy mpMRI according to local standard, and images were interpreted at each site using PI-RADS v2.^24^ Participants provided ≤40 mL first-catch urine without preceding DRE using the FDA-approved Colli-Pee^™^ device, per existing MPS2 non-DRE protocol. Analyte signals were measured per MPS2 non-DRE workflow,^22^ and normalized mean Ct and MPS2-AS values were calculated retrospectively as in the development cohort. All patients underwent ≥12-core transrectal or transperineal systematic biopsy, and patients with PI-RADS≥3 lesions underwent targeted sampling.

### Statistical Analysis

The primary outcome was upgrading to GG≥3 cancer; upgrading to GG≥2 was assessed secondarily. The area under the receiver operating characteristic curve (AUC) and 95% confidence intervals were calculated for discriminative accuracy of MPS2-AS models, PSA, the PCPT risk calculator (PCPTrc),^25^ PSA density (PSAD), and mpMRI. Statistical significance for AUC differences between these testing approaches was determined using the DeLong test. Measures of diagnostic performance (i.e. sensitivity, specificity, negative predictive value [NPV], positive predictive value [PPV]) were calculated retrospectively for each modality. Continuous tests (PSA, PCPTrc, and PSAD) were assessed at the threshold values providing equivalent sensitivity to MPS2-AS, and PSAD was additionally assessed at the conventional threshold of 0.15.^21^ Clinico-demographic variables were measured at the time of enrollment. Prostate volume was obtained from mpMRI or most recent TRUS in patients without mpMRI. Analyses were repeated in clinically-pertinent subgroups: confirmatory and surveillance biopsy, age <65 and ≥65 years, and Black and non-Black race.

Decision curve analysis (DCA) was performed to quantify the net benefit of each testing modality on the decision to perform biopsy compared to i) biopsying all patients and ii) biopsying no patients.^26^ Because risk of upgrading <5% justifies forgoing biopsy and risk >20% justifies performing biopsy in most patients,^2, 27^ these values defined the threshold probability range for GG≥3. Because some patients with GG2 remain AS candidates,^1–3^ a more permissive upper threshold of 30% was used for upgrading to GG≥2. Statistical analyses were performed using R version 4.3.0 and Python version 3.9.20.

We assessed independent and combined diagnostic accuracy of MPS2-AS and mpMRI in post-hoc analysis of patients with both results (N=280). Rates of upgrading associated with negative and positive results were calculated for each test and for concordant and discordant (e.g. MPS2-AS negative/mpMRI positive) results. Finally, we calculated the additive diagnostic value that would have been provided by performing MPS2-AS testing after mpMRI.

## RESULTS

### Validation Cohort

MPS2-AS testing was performed on 338 urine samples, of which 330 yielded valid results (98% valid rate). The external validation cohort thus included 330 men with GG1 PCa presenting for confirmatory or surveillance biopsy (Table 1). Median time from diagnosis to confirmatory biopsy (n=99) was 21 months (IQR 14-40), and median time from prior biopsy was 25 months (IQR 16-44) for patients undergoing surveillance biopsy (n=221). Overall, 280 (85%) patients underwent mpMRI at a median of 2.1 months (IQR 0.9-8.7) prior to index biopsy. Of these, 130 (46%) were classified as PI-RADS 1-2, 42 (15%) as PI-RADS 3, and 108 (39%) as PI-RADS 4-5.

#### GG≥3 Upgrading: Comparative Accuracy and Clinical Consequences of Testing

On study biopsy, 31 (9.4%) patients upgraded to GG≥3, of which 17 (55%) were detected on systematic biopsy only, 6 (19%) on targeted biopsy only, and 8 (26%) on both systematic and targeted biopsy (Table 2A). AUC values (95% CI) for GG≥3 were 0.68 (0.59-0.78) for PSA, 0.70 (0.60-0.79) for PCPTrc, 0.74 (0.65-0.84) for PSAD, and 0.73 (0.63-0.82) for mpMRI, as compared to 0.82 (0.76-0.88) for MPS2-AS (Supplementary Figure 3A). MPS2-AS provided significantly better discrimination for GG≥3 than PSA (p<0.001), PCPTrc (p=0.012), and PSAD (p=0.005) and provided clinically-meaningful improvement relative to mpMRI (0.82 vs. 0.73) that did not meet conventional levels of significance (p=0.10) (Supplementary Table 4A).

Consistent with guidelines,^21^ we used a testing threshold to ensure <10% missed/delayed diagnoses of GG≥3 (i.e. sensitivity ≥90%) and >30% unnecessary biopsies avoided (i.e. specificity >30%). At the validated diagnostic threshold of 11.5%, MPS2-AS testing would have detected 97% of GG≥3 upgrades and avoided 64% of unnecessary biopsies, as compared to avoiding 16% of biopsies using PSA, 17% using PCPTrc, and 23% using PSAD at equal sensitivity. MPS2-AS testing was more sensitive than mpMRI (97% vs. 79-82%) and generally more specific (64% vs. 50-66%) for GG≥3 cancer using either PI-RADS≥3 or PI-RADS≥4 (Table 3A). Applied clinically, MPS2-AS would have missed/delayed detection of 3.2% of GG≥3 cancers, as compared to 18-21% using mpMRI, while MPS2-AS also avoided more unnecessary biopsies.

Accuracy for GG≥3 cancer remained consistent throughout pertinent clinical subgroups (Table 4A). The sensitivity of MPS2-AS was 96-100% across patients undergoing confirmatory and surveillance biopsies, patients <65 and ≥65 years old, and Black and non-Black patients.

#### GG≥2 Upgrading: Comparative Accuracy and Clinical Consequences of Testing

Among the 330 AS patients, 123 (37%) patients upgraded to GG≥2, of which 74 (60%) were detected on systematic biopsy only, 15 (12%) on targeted biopsy only, and 43 (28%) on both systematic and targeted biopsy (Table 2B). AUC values (95% CI) for PSA, PCPTrc, PSAD, and mpMRI were 0.60 (0.53-0.66), 0.64 (0.58-0.70), 0.67(0.61-0.73), and 0.64 (0.58-0.71), respectively, as compared to 0.74 (0.68-0.79) for MPS2-AS (Supplementary Figure 3B). MPS2-AS provided a statistically significant improvement in discrimination over PSA (p<0.001), PCPTrc (p=0.004), PSAD (p=0.005), and mpMRI (p=0.01) (Supplementary Table 4B).

Clinical use of MPS2-AS to determine the need for biopsy would have detected 95% of GG≥2 upgrades while avoiding 34% of unnecessary biopsies (Table 3B). Use of mpMRI to determine the need for biopsy would have missed 35% (PI-RADS≥3) to 45% (PI-RADS≥4) of GG≥2 upgrades. MPS2-AS performance was consistent across subgroups, with 93-97% sensitivity for GG≥2 PCa (Table 4B).

### Decision Curve Analysis

**Figure 1.**
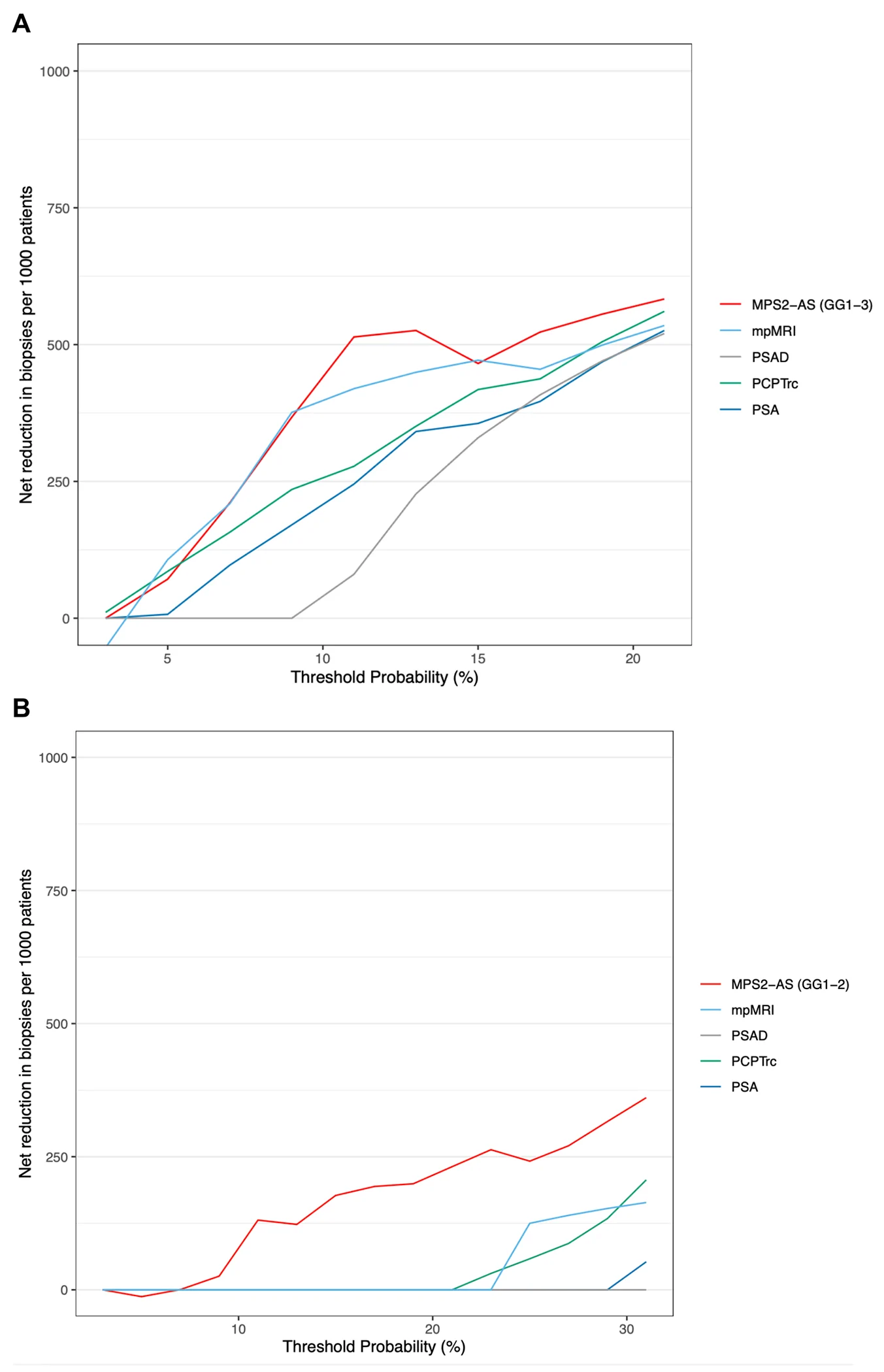
Decision curve analysis plots illustrating the net reduction in biopsies performed per 1000 patients based on pre-biopsy testing with MPS2-AS (red), mpMRI (light blue), PSAD (grey), PCPTrc (green) and PSA (blue) for the outcomes of upgrading to A) GG≥3 and B) GG≥2.

DCA was performed to determine the net clinical benefit of pre-biopsy testing relative to performing biopsy in all patients or no patients. MPS2-AS provided the greatest net reduction in unnecessary biopsies without failing to detect a single additional upgrading event (Figure 1) and the highest net clinical benefit (Supplementary Figure 4) across the vast majority of pertinent threshold probabilities.

### MPS2-AS and mpMRI

#### Combined Accuracy of MPS2-AS and mpMRI

In patients that underwent mpMRI (N=280), concordant positive MPS2-AS and mpMRI provided additive value, as 28% of patients with concordant positive tests upgraded to GG≥3, compared to 23% with positive MPS2-AS alone and 15% with positive mpMRI alone (Table 5A). By contrast, concordant negative results were associated with a 1.1% risk of GG≥3 upgrading, which was higher than the 0.6% risk observed with a negative MPS2-AS alone. When discordant, MPS2-AS was more strongly associated with observed outcomes. GG≥3 upgrading occurred in zero of 68 (0%) patients with a negative MPS2-AS and positive mpMRI, as compared to 11% with positive MPS2-AS and negative mpMRI. Similar associations were observed for the outcome of GG≥2 upgrading (Table 5B).

AUC values (95% CI) for GG≥3 upgrading were 0.73 (0.63-0.82) for mpMRI, 0.80 (0.72-0.88) for mpMRI plus MPS2-AS, and 0.82 (0.76–0.88) for MPS2-AS (Supplementary Figure 5). As such, addition of MPS2-AS to mpMRI improved AUC by 7%, but the addition of mpMRI to MPS2-AS did not improve AUC. For GG≥2 upgrading, AUC values were 0.64 (0.58-0.71) for mpMRI, 0.74 (0.68-0.79) for MPS2-AS, and 0.75 (0.69-0.81 for their combined use.

#### Additive Value of MPS2-AS in Patients with Existing mpMRI

Because an increasing number of patients undergo serial mpMRI during AS, we assessed the diagnostic performance and clinical outcomes of MPS2-AS testing in patients with known mpMRI results (Table 6). In patients with a negative mpMRI, use of a positive MPS2-AS to trigger biopsy would have captured 80% of GG≥3 cancers that would have been missed due to a false negative mpMRI, while still avoiding 74% of unnecessary biopsies. In patients with PI-RADS 3 mpMRI, use of MPS2-AS would have avoided 66% of unnecessary biopsies while maintaining 100% sensitivity for GG≥3 cancer. While patients with PI-RADS 4-5 mpMRI are routinely referred for biopsy, use of MPS2-AS would have avoided 48% of unnecessary biopsies without missing a single diagnosis of GG≥3 cancer (i.e. 100% sensitivity) (Table 6B).

## DISCUSSION

Despite the oncologic safety of AS, repeat biopsies pose a major risk of non-adherence, in addition to discomfort, bleeding, and infectious complications, all of which threaten the safety of this standard-of-care management approach.^13, 28^ There is a pressing need to reduce the number of biopsies required during AS while maximizing timely identification of high-grade cancers that merit treatment. We herein describe multisite external validation of a non-DRE urinary test to inform the need for surveillance biopsies. For the critical diagnosis of GG≥3 cancer, the sensitivity (97%) and specificity (64%) of MPS2-AS indicate that its use in the AS population would avoid 64% of unnecessary AS biopsies while maintaining immediate detection of 97% of GG≥3 upgrades. By contrast, even the most sensitive use of mpMRI would have failed to identify 18% of GG≥3 upgrades, while avoiding fewer unnecessary biopsies (50%). Moreover, mpMRI failed to detect 35% of upgrades to GG≥2, as compared to only 4.9% based on MPS2-AS. Acknowledging the well-established limitations of mpMRI in AS,^9–12, 29, 30^ these data provide an alternative monitoring approach capable of reliably detecting upgrades and ruling out a substantial proportion of unnecessary biopsies.

The current findings from academic and community centers further emphasize the potential drawbacks of mpMRI-based monitoring. Indeed, at one expert academic center, Chu et al observed an NPV of only 79.5% for GG≥2 upgrading.^11^ These findings were corroborated in a larger study of US veterans, in which the NPV of mpMRI was again 79% for patients with GG1 disease undergoing confirmatory or surveillance biopsy.^30^ Our findings are consistent with these and other mpMRI data, revealing NPV of 71% for PI-RADS≥3 in detecting GG≥2 upgrading. Combined, these data clearly demonstrate that a negative mpMRI is not sufficient to rule out the need for surveillance biopsies. By contrast, patients with a negative MPS2-AS had a 0.6% risk of GG≥3 upgrading (99% NPV) and 8.1% risk of GG≥2 upgrading (92% NPV), providing confident rule-out ability in this population. Notably, a negative mpMRI did not provide added NPV beyond a negative MPS2-AS. On the other hand, in patients with a positive MPS2-AS result, a positive mpMRI provided modest additive value, increasing the risk of GG≥3 upgrading from 23% with a positive MPS2-AS alone to 28% with positive MPS2-AS and mpMRI – and from 48% to 53%, respectively, for GG≥2. Taken together, these findings provide a practical clinical testing pathway, whereby a negative MPS2-AS alone can confidently rule-out the need for mpMRI or biopsy, and a positive MPS2-AS can be augmented by existing or repeat mpMRI to guide sampling.

At the same time, these data further emphasize that systematic sampling remains essential in patients undergoing AS biopsies.^9, 31^ We found that systematic biopsy detected the majority of upgrades, including 81% to GG≥3 and 88% to GG≥2, while targeted biopsy captured an additional 19% and 12% of upgrades, respectively. Notably, the proportion of upgrades detected by targeted biopsy was near the lower range of that reported from expert centers.^32, 33^ While these differences could reflect varying provider experience or other factors, these data spanning academic and community centers may better reflect real-world practice.

Regardless, GG≥2 upgrading was captured by either targeted or systematic biopsy in 56% of patients with PI-RADS 4-5 lesions, consistent with detection rates in the literature.^34^ It is thus not expected that higher yield of targeted sampling specifically would substantially impact the observed findings. Importantly, literature describing the lack of informative mpMRI changes during AS suggest that a prior positive mpMRI is likely sufficient to aid in targeting, limiting the need for repeat mpMRI in patients indicated for biopsy based on biomarker testing.^10, 12, 29^ Altogether, the current data add to a large body of evidence revealing that mpMRI is not a reliable primary monitoring tool during AS.^10, 12, 29^ A more practical approach to monitoring would employ a highly-sensitive, objective, non-invasive test to accurately determine the need for repeat biopsy. The current data suggest that MPS2-AS could be highly useful in this role, and by separately providing the likelihood of upgrading to GG≥2 and GG≥3, biopsy decision-making can be further personalized.

The AS population – comprised of men with low-grade, low-volume cancer – has long posed a fundamental challenge to diagnostic tools seeking to detect occult higher-grade disease.^35^ Indeed, it stands to reason that biomarkers developed to distinguish cancer from benign tissue have had limited success in identifying higher-grade cancers among a population of patients known to harbor cancer. While existing serum-based assays have demonstrated an association with upgrading,^15, 16^ none have been shown to provide actionable rule-in or rule-out performance to confidently guide decision-making. Tissue-based genomic classifiers have similarly had little success in predicting upgrading,^36^ potentially due to the variable results obtained depending on the tumor focus submitted for testing.^37, 38^ Freitas and colleagues recently confirmed that molecular testing of the highest volume GG1 core could not distinguish between patients with and without GG≥2 cancer elsewhere in the prostate.^39^ While tissue-based testing remains dependent on sample obtainment and selection, urine provides a comprehensive picture of gene expression from throughout the prostate. Taken with the recent identification of genes uniquely overexpressed in higher-grade cancer relative to GG1,^17^ both biologic and practical factors underlie the observed accuracy of MPS2-AS in the AS population.

The current study has real and perceived limitations. First, this was a multisite study spanning academic and community settings. MRI interpretation was not centralized and was thus subject to site variation. While potentially perceived as a limitation, these are the same interpretations upon which clinical decisions are made for most patients in the US. Importantly, the observed performance of mpMRI is consistent with limited mpMRI data available from combined academic and community settings, supporting their validity.^40, 41^ Second, study inclusion was limited to patients undergoing biopsy as deemed necessary by their treating physician. While this could yield a higher-risk cohort relative to the overall AS population, all testing modalities (i.e. PSA, PCPTrc, PSAD, mpMRI) were evaluated in this same cohort, and MPS2-AS performed favorably. Moreover, a substantial proportion of patients had undergone negative mpMRI, suggesting a broad distribution of risk. Still, these data cannot inform on patients that did not undergo biopsy, just as these data do not inform the comparative performance of MPS2 and mpMRI in the diagnostic setting. Third, MPS2-AS values were calculated retrospectively, and this study was not intended to inform the impact of MPS2-AS on the decision to undergo biopsy. Fourth, the study included a limited number of Black patients, precluding generalization to underrepresented populations. Still, the proportion of Black patients enrolled was consistent with the US population, and, promisingly, MPS2-AS provided 100% sensitivity and NPV for GG≥3 upgrading in Black men. Notably, data were not available on the number of previous biopsies performed or time from diagnosis in patients undergoing surveillance biopsies, which can better risk-stratify among AS patients.^42^ Moreover, histological subtype data, which can provide prognostic information in addition to GG, were not available. Finally, as described, the PPV of MPS2-AS was aided by targeted sampling in 12-19% of upgrades. Still, available data suggest that repeat mpMRI provides new or higher-risk targets in a minority of patients and mpMRI changes are not consistently associated with upgrading,^10, 12, 29^ such that repeat mpMRI can be avoided in most cases in which biomarker testing indicates the need for biopsy. Importantly, the current analysis uniquely characterizes the accuracy of combined biomarker and mpMRI findings, a noted limitation of the existing literature.^21^

## CONCLUSIONS

In this multisite, external validation study among patients on active surveillance, urinary MPS2-AS demonstrated remarkable accuracy to detect upgrading to higher-grade cancer. Importantly, MPS2-AS outperformed mpMRI on direct comparison, providing higher sensitivity for GG≥3 and GG≥2 upgrading and higher specificity for GG≥3. Taken in the context of contemporary clinical practice, these findings support a role for this non-invasive test to guide the need for scheduled biopsies during AS.

## Supplementary Material

1

## Figures and Tables

**Table 1.** Validation Cohort Characteristics

Characteristic	N (%) or Median (IQR)
Total N	330
Median age (IQR), y	69 (63-73)
No. Black race (%)	36 (11)
No. positive family history (%)	56 (17)
No. suspicious DRE (n=323) (%)	17 (5)
Median PSA (IQR), ng/mL	6 (4.4-9.2)
Median prostate volume (IQR)	47 (35-66)
Median PSA density (IQR)	0.12 (0.08-0.2)
	
No. underwent MRI (%)	280 (85)
PI-RADS 1-2 (% of mpMRI)	130 (46)
PI-RADS 3	42 (15)
PI-RADS 4	73 (26)
PI-RADS 5	35 (13)
	
Biopsy indication	
Confirmatory biopsy	99 (30)
Surveillance biopsy	221 (67)
Not reported	10 (3)
	
Biopsy results	
Benign/Grade Group 1	207 (63)
Grade Group 2	92 (28)
Grade Group 3	21 (6)
Grade Group 4	8 (2)
Grade Group 5	2 (1)
	
Grade Group ≥2	123 (37)
Grade Group ≥3	31 (9.4)

Abbreviations: IQR, interquartile range; DRE, digital rectal exam; PSA, prostate-specific antigen; mpMRI, multi-parametric magnetic resonance imaging; PI-RADS, Prostate Imaging Reporting and Data System.

**Table 2.** Detection of Upgrading by Systematic and Targeted Biopsy. Values represent N (%) of upgrades unless otherwise indicated.

Upgraded N (% of cohort)	Detected on SBx	Detected on TBx	Detected on SBx Only	Detected on TBx Only	Detected on SBx & TBx
***A. GG1 to GG≥3***					
31 (9.4)	25 (81)	14 (45)	17 (55)	6 (19)	8 (26)
***B. GG1 to GG≥2***					
123 (37)	108 (88)	49 (40)	74 (60)	15 (12)	34 (28)

Abbreviations: SBx, systematic biopsy; TBx, targeted biopsy; GG, Grade Group.

**Table 3.** Diagnostic Accuracy and Clinical Consequences of MPS2-AS, PSA, PCPTrc, PSAD, and mpMRI for Prediction of Upgrading in the Validation Cohort

	Threshold	Sens (%)	Spec (%)	NPV (%)	PPV (%)	Potentially missed/delayed upgrading (%)	Unnecessary Bx potentially avoided per 1000 patients
***A. GG1 to GG≥3***							
MPS2-AS	11.5%	97	64	99	22	3.2	635
PSA	3.7	97	16	98	11	3.2	164
PCPTrc	6.0%	97	17	98	11	3.2	171
PSAD	0.08	97	23	99	12	3.2	234
PSAD	0.15	65	65	95	16	35	652
mpMRI	PI-RADS ≥3	82	50	96	15	18	496
	PI-RADS ≥4	79	66	97	20	21	659
***B. GG1 to GG≥2***							
MPS2-AS	11.5%	95	34	92	46	4.9	338
PSA	2.9	95	12	80	39	4.9	116
PCPTrc	5.1%	95	13	82	39	4.9	130
PSAD	0.05	95	12	81	39	4.9	121
PSAD	0.15	54	72	72	53	46	720
mpMRI	PI-RADS ≥3	65	54	71	47	35	538
	PI-RADS ≥4	55	72	72	56	45	719

Abbreviations: MPS2-AS, MyProstateScore 2.0 - Active Surveillance; PSA, prostate-specific antigen; PCPTrc, Prostate Cancer Prevention Trial Risk Calculator; PSAD, prostate specific antigen density; mpMRI, multiparametric magnetic resonance imaging; Sens, sensitivity; Spec, specificity; NPV, negative predictive value; PPV, positive predictive value; Bx, biopsy; GG, Grade Group; PI-RADS, Prostate Imaging Reporting and Data System.

**Table 4.** Clinical Performance Measures of MPS2-AS in Pertinent Subgroups at 11.5% Threshold

Subgroup	N	Upgraded	Sens (%)	Spec (%)	NPV (%)	PPV (%)
***A. GG1 to GG≥3***						
Confirmatory Bx	99	4 (4%)	100	56	100	9
Surveillance Bx	221	26 (12%)	96	68	99	29
Age < 65 years	108	6 (6%)	100	68	100	15
Age ≥ 65 years	222	25 (11%)	96	61	99	24
Non-Black Race	294	26 (9%)	96	65	99	21
Black Race	36	5 (14%)	100	48	100	24
***B. GG1 to GG≥2***						
Confirmatory Bx	99	36 (36%)	97	27	94	43
Surveillance Bx	221	85 (39%)	94	38	91	48
Age < 65 years	108	30 (28%)	97	37	97	37
Age ≥ 65 years	222	93 (42%)	95	32	89	50
Non-Black Race	294	109 (37%)	95	35	93	46
Black Race	36	14 (39%)	93	27	86	45

Abbreviations: MPS2-AS, MyProstateScore 2.0 - Active Surveillance; Sens, sensitivity; Spec, specificity; NPV, negative predictive value; PPV, positive predictive value; GG, Grade Group; Bx, biopsy; PSA, prostate-specific antigen.

**Table 5.** Individual and Combined Accuracy of MPS2-AS and mpMRI for Detection of Upgrading

Subgroup	PI-RADS	MPS2-AS ≤11.5%	MPS2-AS >11.5%	Total
**A1. GG1 to GG≥3**	1-2	1.1% (1/93)	11% (4/37)	3.8% (5/130)
	3-5	0% (0/68)	28% (23/82)	15% (23/150)
	**Total**	0.6% (1/161)	23% (27/119)	10% (28/280)
**A2. GG1 to GG≥2**	1-2	9.1% (4/44)	40% (34/86)	29% (38/130)
	3-5	5.6% (1/18)	53% (70/132)	47% (71/150)
	**Total**	8.1% (5/62)	48% (104/218)	39% (109/280)

Abbreviations: MPS2-AS, MyProstateScore 2.0 - Active Surveillance; mpMRI, multiparametric magnetic resonance imaging; PI-RADS, Prostate Imaging Reporting and Data System; GG, Grade Group.

**Table 6.** Performance Characteristics of MPS2-AS in Patients with Known PI-RADS Classification

			MPS2-AS Performance in PI-RADS Strata				Potentially missed/delayed upgrading (%)	Unnecessary Bx potentially avoided per 1000 patients
Subgroup	N	Upgraded	Sens (%)	Spec (%)	NPV (%)	PPV (%)		
***A. GG1 to GG≥3***								
PI-RADS 1-2	130	5 (4%)	80	74	99	11	20	736
PI-RADS 3	42	1 (2%)	100	66	100	7	0	659
PI-RADS 4-5	108	22 (20%)	100	48	100	33	0	477
***B. GG1 to GG≥2***								
PI-RADS 1-2	130	38 (29%)	89	43	91	40	11	435
PI-RADS 3	42	11 (26%)	100	29	100	33	0	290
PI-RADS 4-5	108	60 (56%)	98	17	89	60	1.7	167

Abbreviations: MPS2-AS, MyProstateScore 2.0 - Active Surveillance; PI-RADS, Prostate Imaging Reporting and Data System; Sens, sensitivity; Spec, specificity; NPV, negative predictive value; PPV, positive predictive value; Bx, Biopsy; GG, Grade Group.
