# Supplementary material for: Non-Invasive Urine Test Predicts Grade Group Upgrading in Patients on Active Surveillance for Prostate Cancer: Multisite Validation and Comparison with MRI
Source: J Urol. Author manuscript; Available in PMC 2026 Jul 9. (PMC13345705; doi:10.1097/JU.0000000000005095)
Supplement: Supplementary file 1 [file NIHMS2178140-supplement-1.pdf]

SUPPLEMENT

Non-Invasive Urine Test Predicts Grade Group Upgrading in Patients on Active Surveillance for Prostate Cancer: Multisite Validation and Comparison with MRI

Tosoian, Meyers, Moore et al.

Development Cohort

The development cohort included 761 patients of median age 63 (IQR 58-68) and median PSA 5.6 ng/ml (IQR 4.6-7.2) who presented to the University of Michigan for diagnostic TRUS-guided prostate biopsy due to elevated PSA (3-10 ng/ml) from January 2008 through December 2020.[1] All patients underwent ≥12 core biopsy without pre-biopsy imaging. Pre-biopsy urine was prospectively-collected under a National Cancer Institute (NCI) Early Detection Research Network (EDRN) protocol. Clinical and demographic characteristics of the cohort are included in Supplementary Table 1.

Model Development

We developed distinct models to predict cancer upgrading from GG1 to GG≥3 (MPS2-AS GG1-3 model) and GG1 to GG≥2 (MPS2-AS GG1-2 model). Applying the MPS2 18-gene panel in the development cohort, supervised and unsupervised feature engineering methods were employed, such as inclusion of pairwise interactions, principal component analysis (PCA), linear discriminant analysis (LDA), multivariate adaptive regression splines (MARS), correlation-based filtering, and a primary focus on recursive feature elimination (RFE) and coefficient-based filtering. PSA density (PSAD) was locked into all model candidates *a priori* based on predictive value for high-grade cancer.[2] For high reproducibility and ease of interpretation, candidates were limited to those using linear algorithms. For candidate evaluation, hyperparameters were tuned via grid search, and performance was evaluated using repeated k-fold (10 folds, 5 repeats) cross-validation AUC (CV-AUC). For the top candidate GG1-3 model, CV-AUC ranged from 0.71-0.80. For the top candidate GG1-2 model, CV-AUC ranged from 0.74-0.89.

Given that the development cohort was not an active surveillance cohort while the validation cohort was, we prioritized simplicity during the final model selection process to better ensure generalizability. More specifically, we identified a set of core features among model candidates with the highest CV-AUC values. Among candidates limited to these features, we observed little discrepancy between the CV-AUC values of the top five candidates. Thus, to ensure generalizability, we analyzed the calibration plots of these top candidates in the validation cohort and made final selections based on the model that best matched predicted probabilities with observed event rates across the range of predicted risk.[3] In final, regularized logistic regression models using a generalized linear model (GLM) framework were selected for both the GG≥3 and GG≥2 models.

Supplementary Table 1. Development Cohort Characteristics

Cohort Characteristic	N (%) or Median (IQR)
Total N	761
Median age (IQR)	63 (58-68)
No. Black race (%)	33 (4.3)
No. positive family history (%)	206 (27)
No. suspicious DRE (%)	104 (14)
Median PSA (IQR), ng/mL	5.6 (4.6-7.2)
Median prostate volume (IQR)	48 (36-66)
Median PSA density (IQR)	0.12 (0.08-0.16)
No. previous negative biopsy (%)	163 (21)
Biopsy results	
Benign	362 (48)
GG1	106 (14)
GG2	168 (22)
GG3	72 (9.5)
GG4	25 (3.3)
GG5	28 (3.7)

Abbreviations: IQR, interquartile range; DRE, digital rectal exam; PSA, prostate-specific antigen; GG, Grade Group.

Supplementary Table 2. Biomarkers Included in MPS2-AS Models

**GG1-3 (5)**	**GG1-2 (9)**
KLK3	KLK3
PCA3	PCA3
PCAT14	PCAT14
TRGV9	TRGV9
OR51E2	CAMKK2
	SCHLAP1
	SPON2
	T2:ERG
	TFF3

Abbreviations: MPS2-AS, MyProstateScore 2.0 - Active Surveillance; GG, Grade Group; T2:ERG, TMPRSS2:ERG fusion.

Supplementary Table 3. Calculated and Observed Sample Sizes for Validation

MPS2-AS Model	Total (N)		Cases (N)		Controls (N)	
	Estimated Sample Size Required	Validation Cohort	Estimated Sample Size Required	Validation Cohort	Estimated Sample Size Required	Validation Cohort
GG1-3	297	330	28	31	269	299
GG1-2	94	330	35	123	59	207

[a] Sample size estimation designed for a rule-out test with a continuous biomarker was employed,[4] where minimum acceptable sensitivity and specificity values are defined in the calculation (e.g. minimal sensitivity = 90%, minimal specificity = 30%, alpha = 0.05, power = 75%, alternative false positive rate = 40%). This yielded an estimated sample size requirement of 297 for GG1-3 (including ≥28 cases), and 94 for GG1-2 (≥35 cases). Observed sample sizes exceeded the sample sizes required.

Abbreviations: MPS2-AS, MyProstateScore 2.0 - Active Surveillance; GG, Grade Group.

Supplementary Table 4. Area Under the Curve Comparisons for GG≥3 and GG≥2 Upgrading

	AUC (95% CI)	P-value vs. MPS2-AS
A. GG1 to GG≥3		
MPS2-AS	0.82 (0.76-0.88)	
PSA	0.68 (0.59-0.78)	<0.001
PCPTrc	0.70 (0.60-0.79)	0.012
PSAD	0.74 (0.65-0.84)	0.005
mpMRI	0.73 (0.63-0.82)	0.10
B. GG1 to GG≥2		
MPS2-AS	0.74 (0.68-0.79)	
PSA	0.60 (0.53-0.66)	<0.001
PCPTrc	0.64 (0.58-0.70)	0.004
PSAD	0.67 (0.61-0.73)	0.005
mpMRI	0.64 (0.58-0.71)	0.01

[a] Formal AUC comparisons between MPS2-AS and each of PSA, PCPTrc, PSAD, and mpMRI were performed using the DeLong test for correlated data for upgrading to GG≥3 (Panel A) and to GG≥2 (Panel B). For comparisons involving mpMRI, analysis was performed in the cohort of men with mpMRI data (N=280). P-values <0.05 denotes a statistically significant improvement in discrimination for the MPS2-AS model.

Abbreviations: GG, Grade Group; AUC, area under the curve; CI, confidence interval; MPS2-AS, MyProstateScore 2.0 - Active Surveillance; PSA, prostate-specific antigen; PCPTrc, Prostate Cancer Prevention Trial Risk Calculator; PSAD, prostate-specific antigen density; mpMRI, multiparametric magnetic resonance imaging.

Supplementary Figure 1. Calibration curves for detection of upgrading to GG≥3 (blue) and GG≥2 (orange) reflect good calibration of predicted and observed risk.

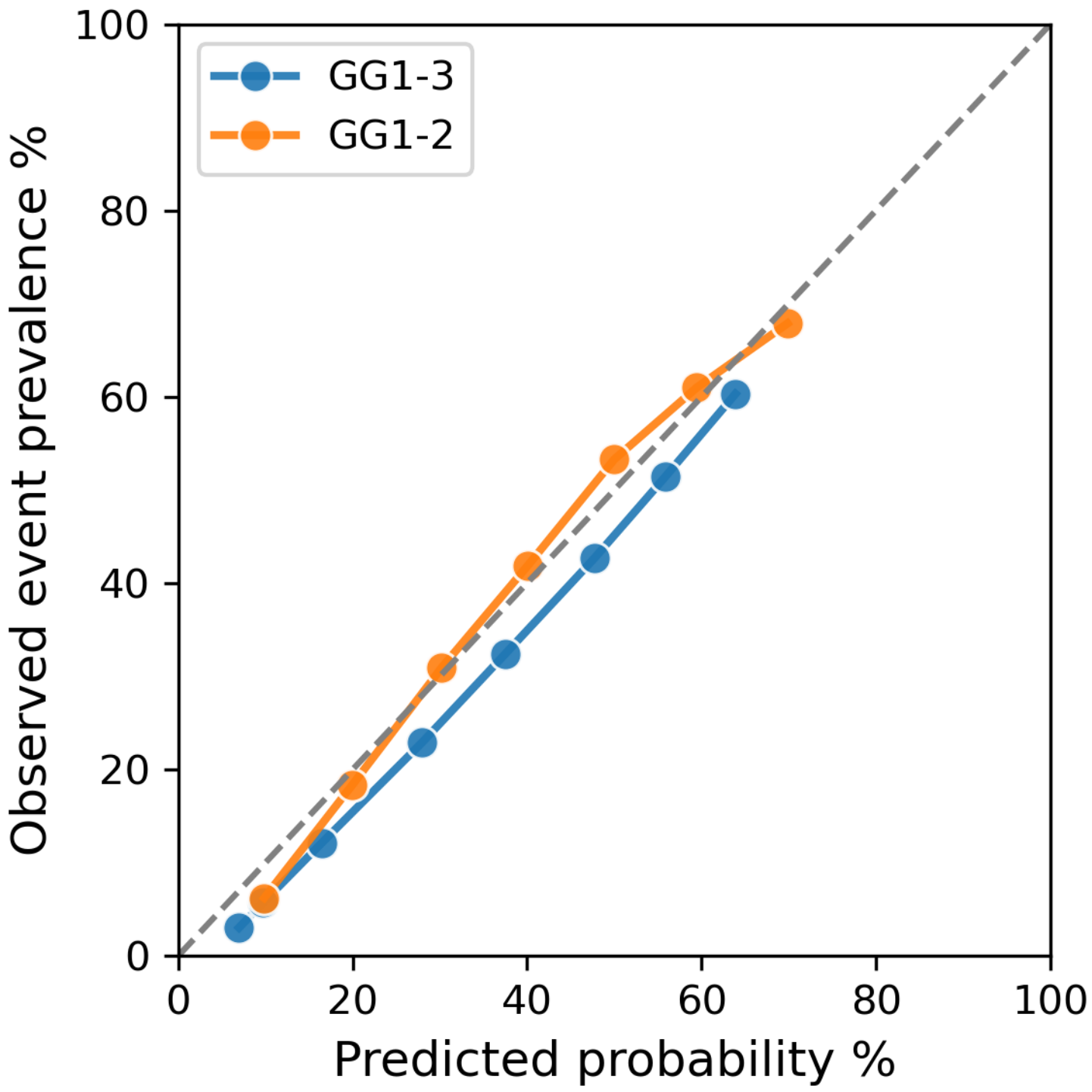

Supplementary Figure 2. The study cohort included patients undergoing active surveillance for GG1 cancer. Patients undergoing diagnostic biopsy or taking 5-ARI medications were excluded.

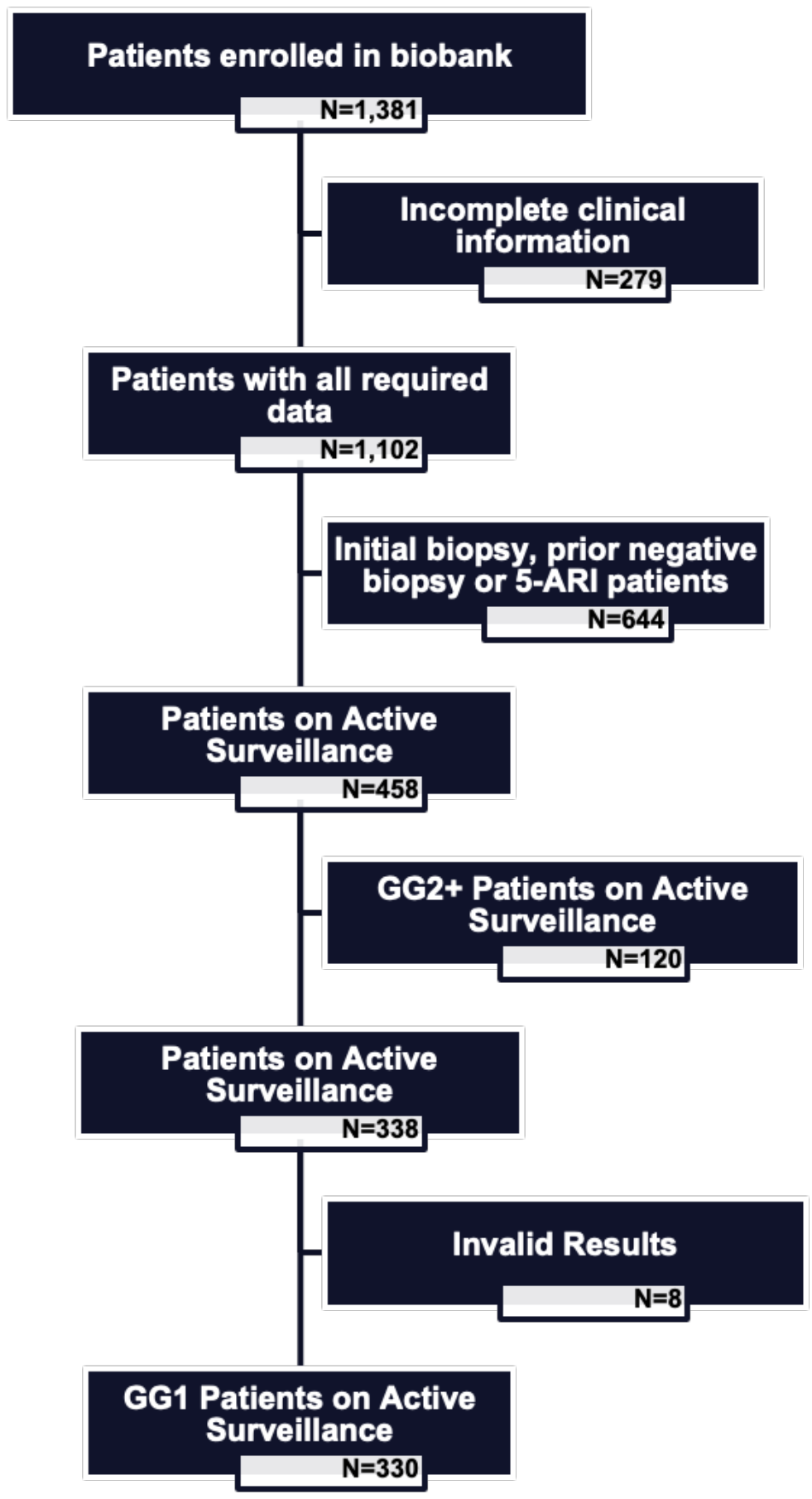

Supplementary Figure 3. Receiver-operating characteristic curves and areas under the curve (AUC) for upgrading for external validation cohort with 95% confidence intervals [95% CI] for upgrading to A) GG≥3 and B) GG≥2 based on MPS2-AS (red), mpMRI (light blue), PSAD (grey), PCPTrc (green) and PSA (blue). The dashed horizontal line reflects sensitivity at the threshold value.

A

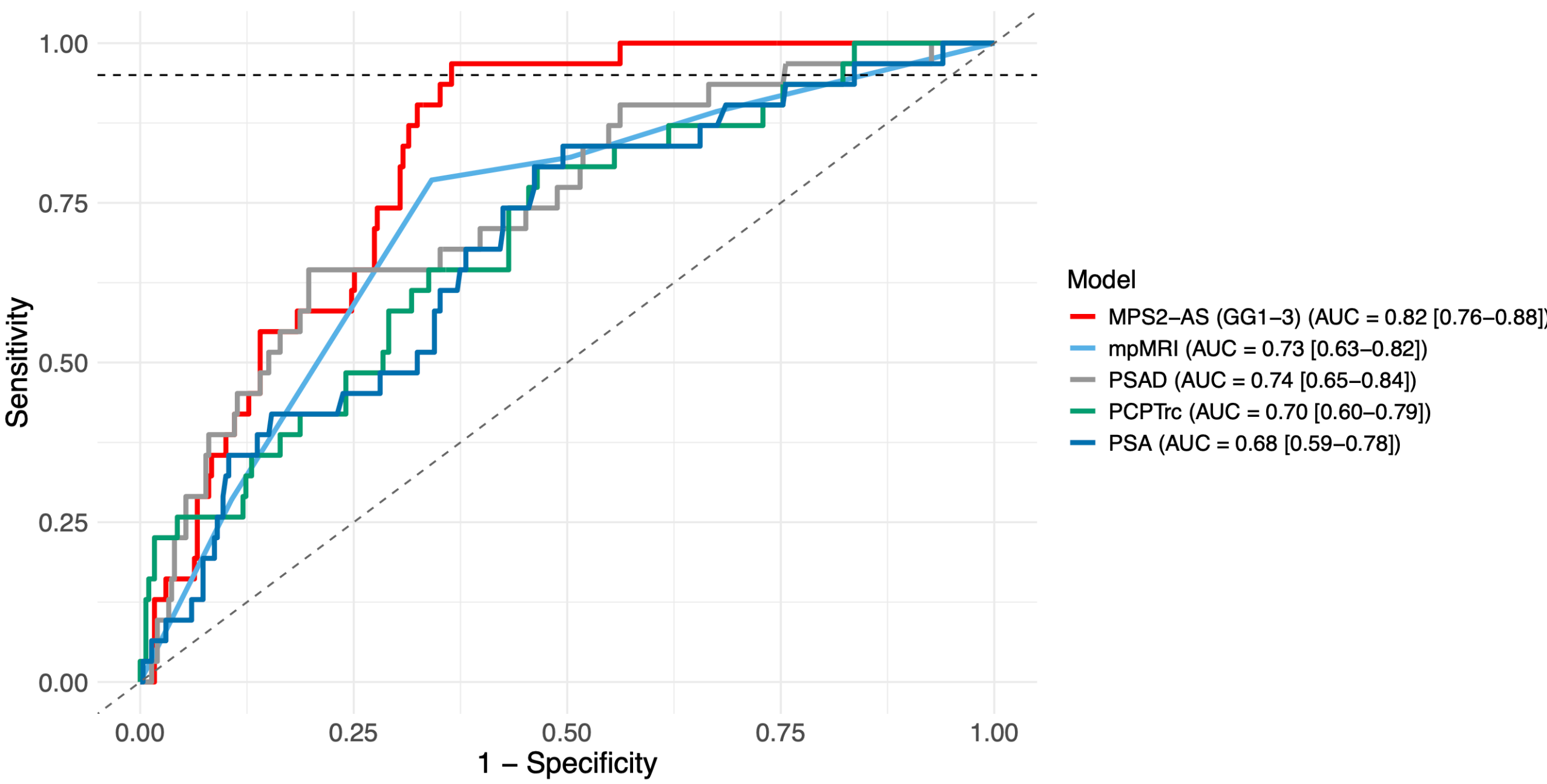

B

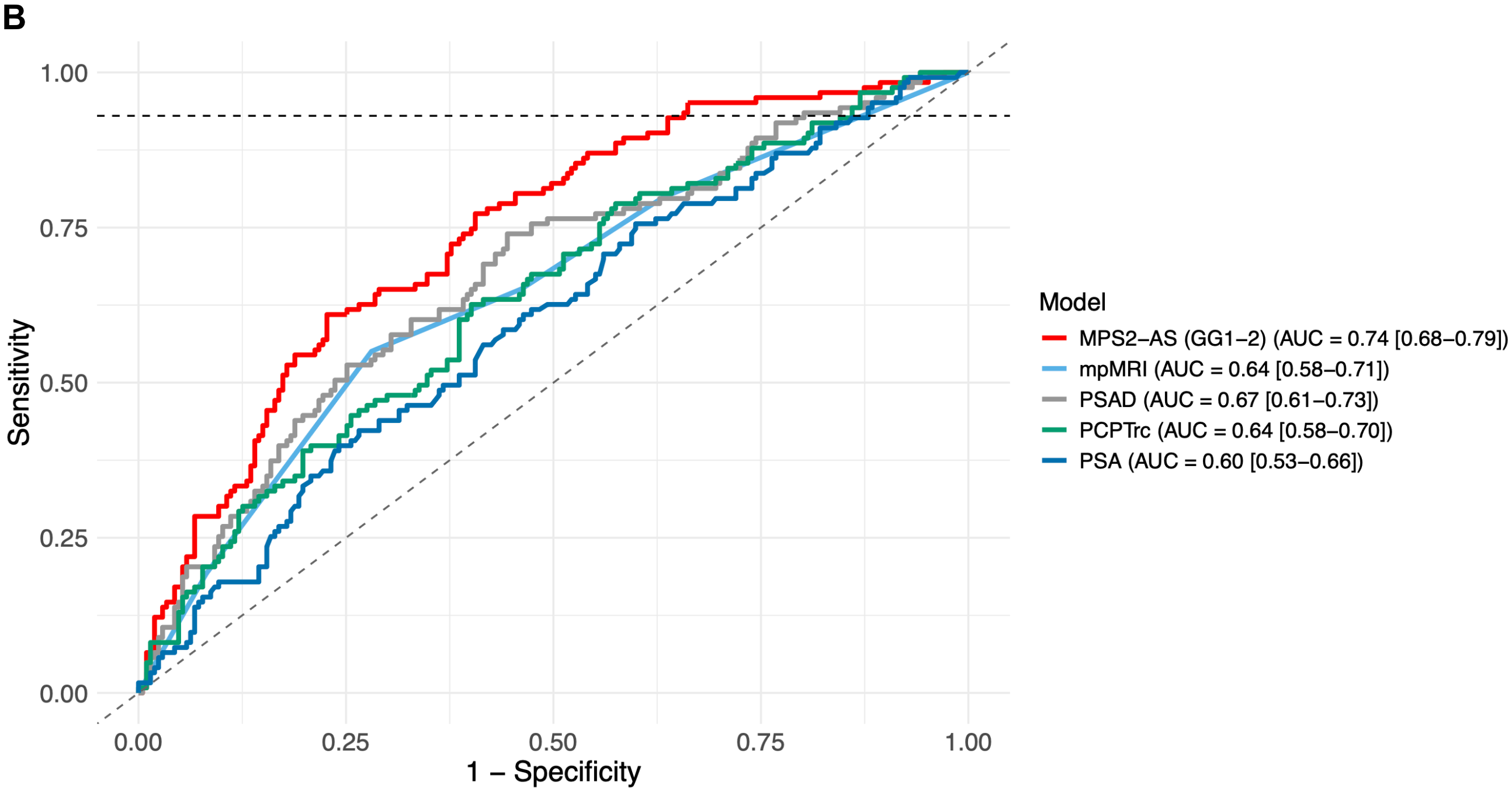

Supplementary Figure 4. Decision Curve Analysis (DCA) plots illustrating the net clinical benefit based on pre-biopsy testing with MPS2-AS (red), mpMRI (light blue), PSAD (grey), PCPTrc (green) and PSA (blue) as compared to biopsying all patients (black) and biopsying no patients (dashed black) for the outcomes of upgrading to A) GG≥3 and B) GG≥2.

A

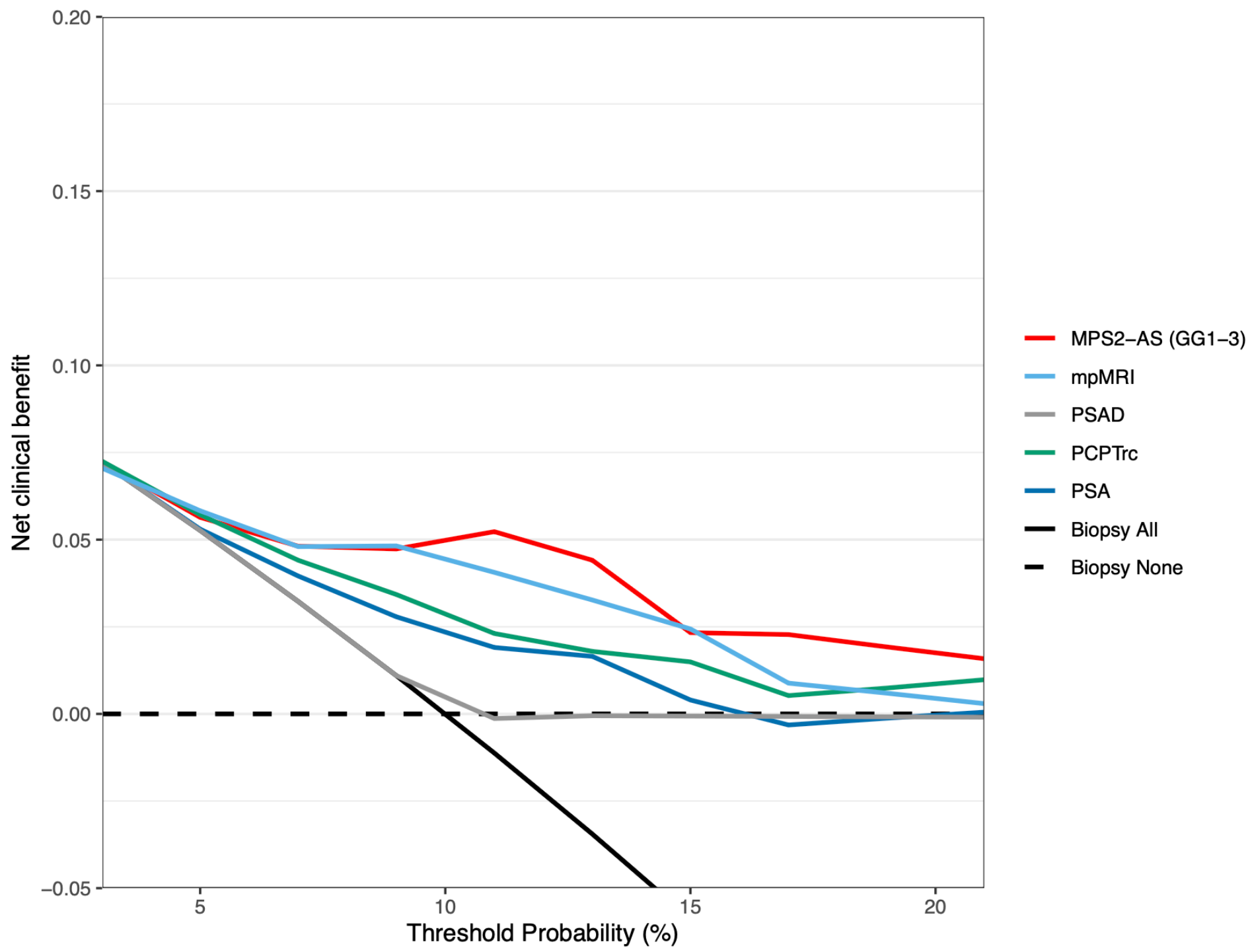

B

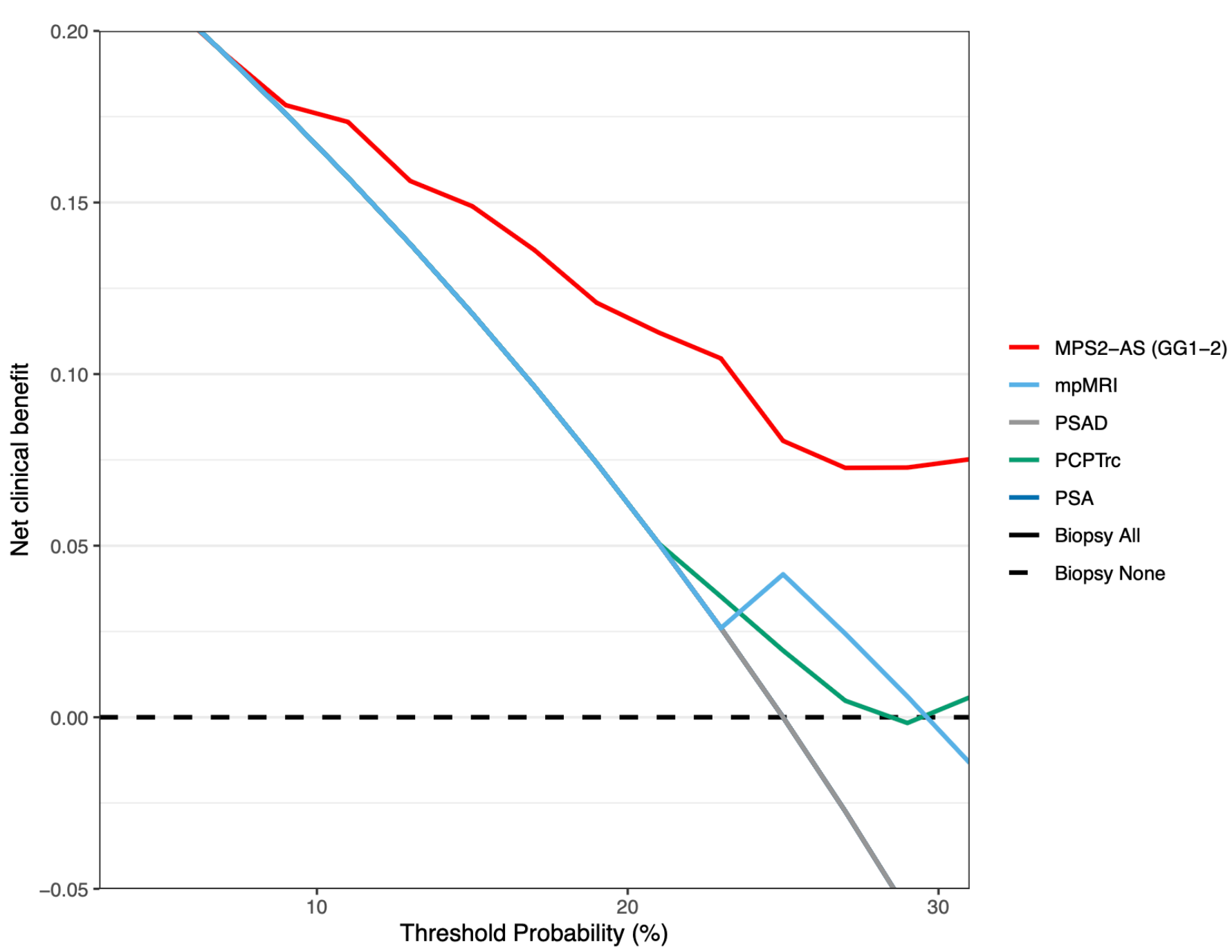

Supplementary Figure 5. Receiver-operating characteristic curves and areas under the curve (AUC) for external validation cohort with 95% confidence intervals [95% CI] for upgrading to A) GG≥3 and B) GG≥2 based on MPS2-AS (red), MPS2-AS plus mpMRI (purple), and mpMRI (light blue). The dashed horizontal line reflects sensitivity at the threshold value.

A

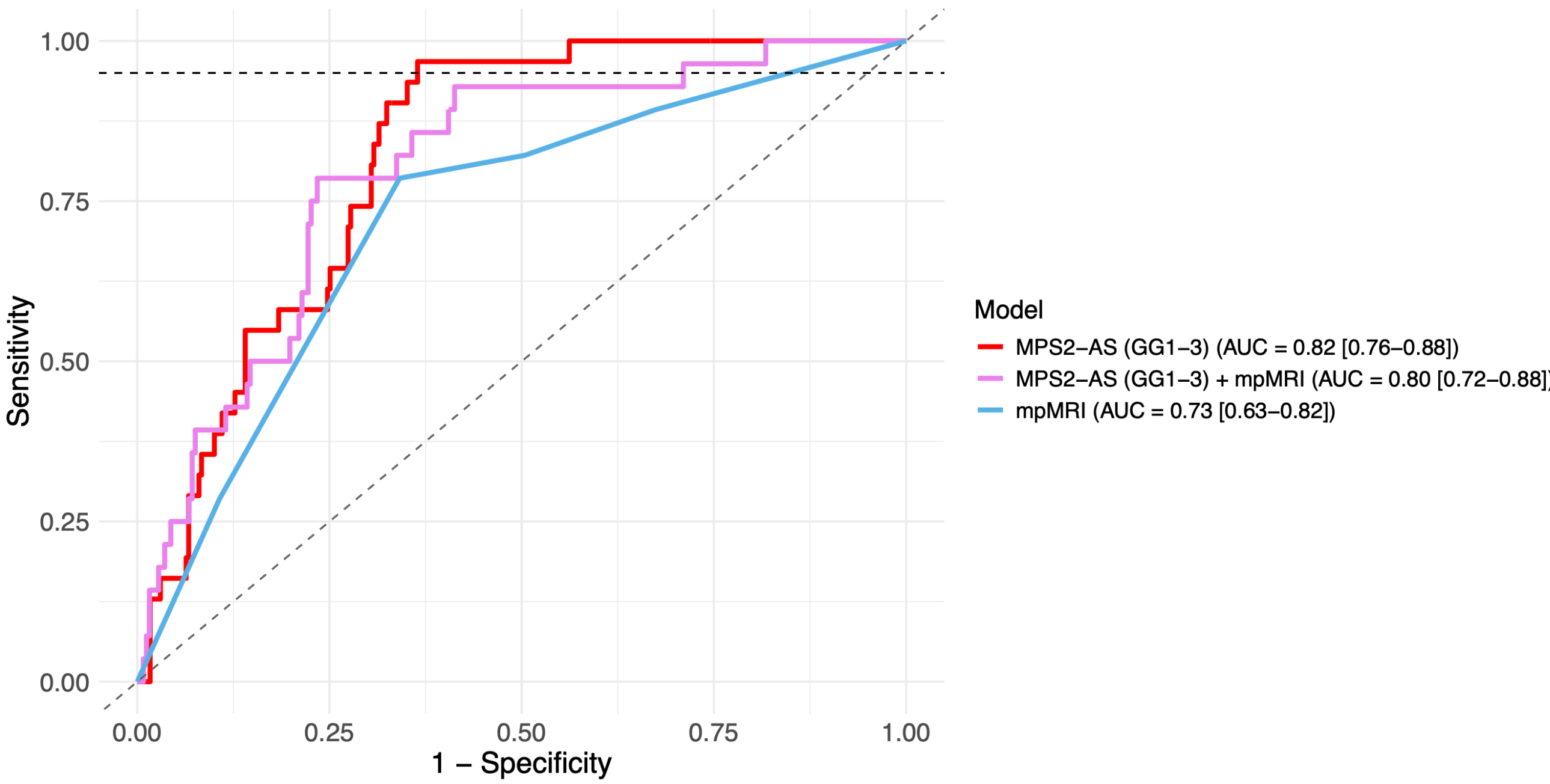

B

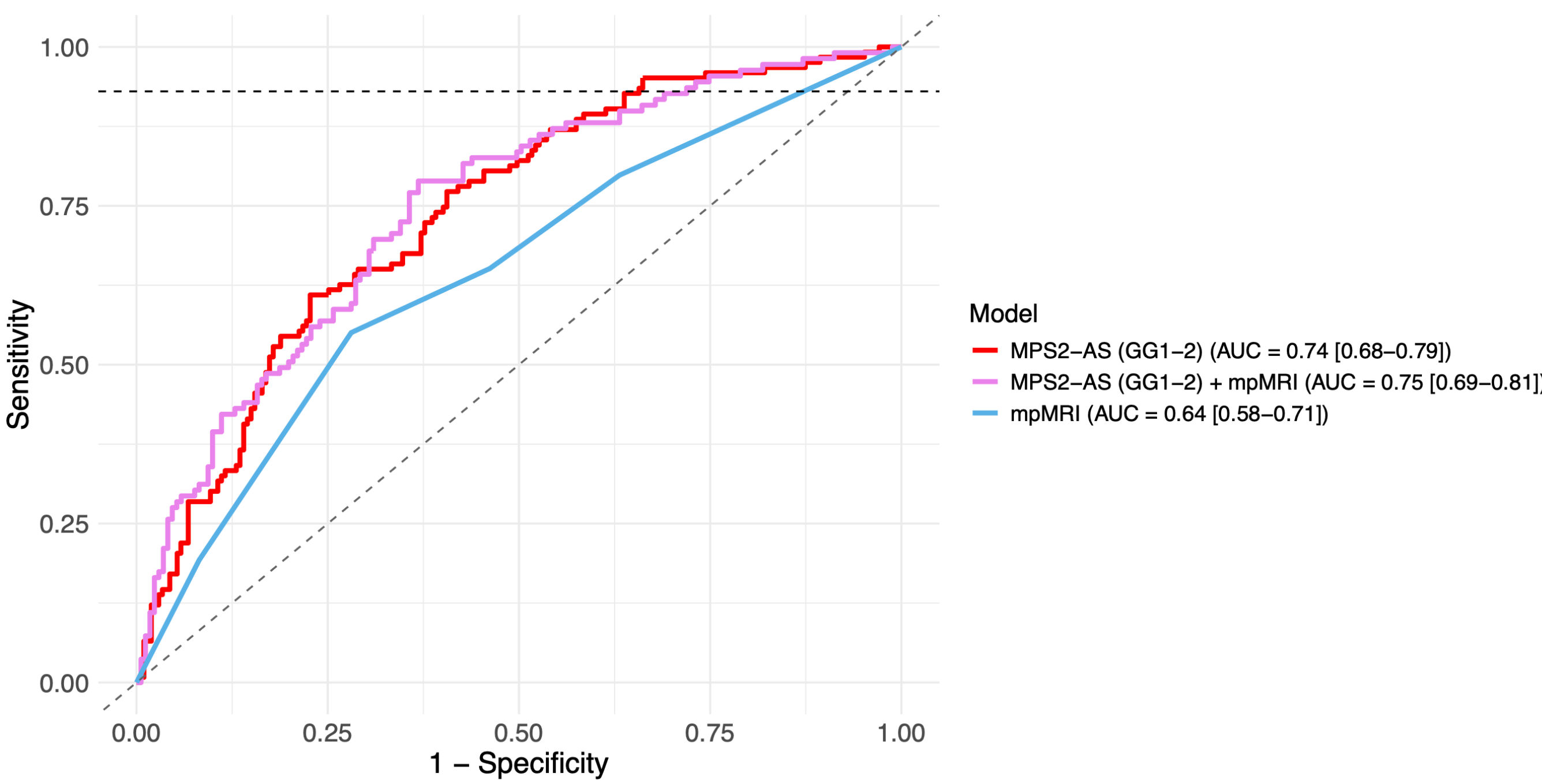

REFERENCES

1. Tosoian, J. J., Zhang, Y., Xiao, L. et al.: Development and Validation of an 18-Gene Urine Test for High-Grade Prostate Cancer. JAMA Oncol, **10:** 726, 2024
2. Wang, S., Kozarek, J., Russell, R. et al.: Diagnostic Performance of Prostate-specific Antigen Density for Detecting Clinically Significant Prostate Cancer in the Era of Magnetic Resonance Imaging: A Systematic Review and Meta-analysis. Eur Urol Oncol, **7:** 189, 2024
3. Ren, Q., Gu, J., Lu, N.: Development and validation of a combined diagnostic model for prostate cancer integrating MRI parameters with p504s, CK5/6, and Ki-67 expression. Open Life Sci, **21:** 20251244, 2026
4. Pepe, M. S., Feng, Z., Janes, H. et al.: Pivotal evaluation of the accuracy of a biomarker used for classification or prediction: standards for study design. J Natl Cancer Inst, **100:** 1432, 2008
